# The Continental Divide: Anti-TNF Use in Pediatric IBD Is Different in North America Compared to Other Parts of the World

**DOI:** 10.1155/2018/3190548

**Published:** 2018-06-12

**Authors:** Peter C. Church, Jeffrey Hyams, Frank Ruemmele, Lissy de Ridder, Dan Turner, Anne M. Griffiths

**Affiliations:** ^1^Division of Gastroenterology, Hepatology & Nutrition, The Hospital for Sick Children, Department of Paediatrics, University of Toronto, Toronto, Canada; ^2^Division of Digestive Diseases, Hepatology, and Nutrition, Connecticut Children's Medical Center, Hartford, CT, USA; ^3^University of Connecticut School of Medicine, Farmington, CT, USA; ^4^Université Sorbonne Paris Cité, Université Paris Descartes, and Assistance Publique-Hôpitaux de Paris, Hôpital Necker-Enfants malades, Service de Gastroentérologie Pédiatrique, Paris, France; ^5^Department of Pediatric Gastroenterology, The Erasmus MC Sophia Children's Hospital, Rotterdam, Netherlands; ^6^The Juliet Keidan Institute of Pediatric Gastroenterology and Nutrition, Shaare Zedek Medical Center, The Hebrew University of Jerusalem, Jerusalem, Israel; ^7^Sick Kids Inflammatory Bowel Disease Centre, The Hospital for Sick Children, Toronto, Canada

## Abstract

**Background and Aims:**

Use of anti-TNF therapies varies internationally. As an initiative of the international Pediatric IBD Network (PIBDNet), we compared global pediatric IBD anti-TNF practice patterns.

**Methods:**

Physicians were surveyed about anti-TNF use in Crohn's disease (CD) and ulcerative colitis (UC). Chi-squared, independent samples Mann–Whitney* U*, or related samples Wilcoxon signed rank tests were used to compare groups.

**Results:**

344 physicians treating pediatric IBD responded from 43 countries (54% North America, 29% Europe, 6% Oceania, 6% Asia, 3% Africa, and 2% South America). Respondents treated a median 40 IBD patients. CD was more commonly treated with anti-TNF than UC (40% vs. 10%, p<0.001). North Americans more often used anti-TNF (median 50% vs. 30%, p<0.001) and before immunomodulator (80% vs. 35% CD, p<0.001; 76% vs. 43% steroid-dependent UC, p<0.001). Anti-TNF monotherapy was more common in North America. Anti-TNF in combination with methotrexate, instead of thiopurine, characterized North American practices. North Americans more often continued immunomodulator indefinitely and less often adhered to standard infliximab induction dosing. Access limitations were more common outside North America and Europe for both CD (67% vs. 31%, p<0.001) and UC (62% vs. 33%, p<0.001).

**Conclusions:**

Anti-TNF use in North America varies significantly from elsewhere.

## 1. Introduction

Anti-TNF antibodies are highly effective therapies for treating pediatric inflammatory bowel disease (IBD). Industry-initiated multicentre clinical trials involving open-label induction followed by randomized dose-ranging maintenance therapy support the efficacy of infliximab and adalimumab in luminal inflammatory pediatric Crohn's disease (CD) and of infliximab in pediatric ulcerative colitis (UC) [1–3]. The ability of anti-TNF therapy to heal the intestine, thereby improving long-term outcomes, makes these biologics particularly important for children [4, 5], among whom IBD is occurring increasingly frequently globally [6]. As an initiative of the international Pediatric IBD Network (PIBDNet), we aimed to explore regional differences in anti-TNF therapy practice patterns in pediatric IBD. Examining the variation in anti-TNF use among children internationally is important for understanding the timing, dosing, and attention to maintaining durability of response. Understanding regional differences in practice could aid in interpretation of studies reporting outcomes and safety data which sometimes vary by country. Also, allocated resources depend on local needs and existing routine practices. Identifying differences in practice is the first step to studying the effect these differences have on outcomes of interest, with the goal of optimizing care among children with long lives ahead during which effective IBD therapy is required.

## 2. Materials and Methods

PIBDNet was established in 2015 as a worldwide network of pediatric IBD specialists with the overall aim of improving outcomes in children with IBD globally, as new therapies emerge, through facilitation of investigator-initiated and industry-sponsored pediatric clinical trials, monitoring of drug safety, and advocacy with regulatory bodies. Membership is open to all physicians, who treat children and adolescents with IBD.

An electronic survey (Supplemental Digital Content) was developed by the PIBDNet executive through an iterative, consensus process and sent to all PIBDNet members. Other potential respondents were ascertained using national membership lists provided by PIBDNet members (from Belgium, Brazil, Canada, the Czech Republic, Finland, France, Italy, Malaysia, Portugal, and Singapore). In addition, subscribers to the Pediatric GI Bulletin Board hosted at the University of Vermont were included. Participants were asked to complete the survey if they currently treated any number of children with IBD.

There were 8 questions related to demographics. Respondents identified their country and type of practice (university-based vs. community-based) and number of years in practice. We used the “composition of macro geographical (continental) regions” database maintained by the United Nations Statistics Division to associate each chosen country with its corresponding continent [7]. We characterized the respondents' patient population by number and percentage of current patients actively being followed with IBD, the proportion of CD to UC, and the age of the oldest IBD patient ever treated.

There were 23 questions which explored practice patterns in CD including percentage of patients treated with anti-TNF therapy (separating infliximab (IFX) and adalimumab (ADA) for both luminal and perianal fistulizing CD) and percentage of time anti-TNF therapy is used first line without a trial of immunomodulator (IM). With respect to luminal inflammatory CD, respondents were asked how often an IM is started with IFX or ADA in different scenarios, choice of IM (methotrexate (MTX) vs. thiopurines), and for how long they continue concomitant IM. Next, we asked how often standard induction dosing regimens were followed for IFX (5mg/kg/dose at weeks 0, 2, and 6) and ADA (160mg/80mg followed by 40mg every other week, if >40kg; 80mg/40mg followed by 20mg every other week, if <40kg) and we explored how the dosing regimens are modified. Respondents were asked how often anti-TNF therapy was discontinued for patients with luminal inflammatory CD in continuous remission and how often anti-TNF therapy was started after surgical resection as postoperative preventive therapy.

There were 14 questions which explored ulcerative colitis (UC). We examined the percentage of patients with UC treated with anti-TNF therapy (IFX vs. other) and how often it was used without a trial of IM in steroid-dependent patients. As with CD, we explored deviation from the standard IFX induction regimen for patients with both steroid-refractory and steroid-dependent UC. Respondents reported their use of combination IM (MTX vs. thiopurine) in IM-naïve patients and after IM failure and the usual duration of combination therapy. Finally, we asked about stopping anti-TNF therapy for patients with UC in continuous remission.

We included 5 questions about routine safety testing before and during anti-TNF therapy. Finally, we explored access limitations (3 questions).

The survey was open to responses between September and November 2015. Potential respondents were invited by email to complete an electronic survey hosted on the REDCap platform [8]. After the initial invitation, 4 further email reminders were sent.

### 2.1. Analysis

Results are presented as N (%), or median and interquartile range (IQR) as appropriate. To facilitate analyses, we planned to categorize respondents into 6 regions: North America, Europe, Asia, Oceania, Africa, and South America. However, there were relatively few respondents from the last 4 continents, and so they were combined. Our main analysis thus compared respondents from North America, Europe, and elsewhere. Additional subanalyses included comparisons of respondents from Canada compared to the USA, those in university-based practice vs. community-based practice, and those early in practice (<10 years) vs. later in practice (≥10 years).

Differences between groups were assessed by Chi-square or Fisher's exact test (depending on cell sizes) for categorical variables and Mann–Whitney* U* test (independent samples) or related samples Wilcoxon signed rank test (paired samples) for continuous variables.

Statistical analyses were performed using SPSS Statistics for Windows, Version 23.0 (Armonk, NY: IBM Corp). In all analyses, p values <0.05 were considered significant.

## 3. Results

PIBDNet members contributed 404 names. Another 1396 potential respondents, representing at least 52 countries, came from the pediatric gastroenterology bulletin board, after eliminating gastroenterology fellows/trainees, surgeons, pathologists, nurses, nurse practitioners, or dietitians. This left a maximum of 1800 potential respondents. However, several of these would be practicing primarily hepatology, pancreatology, and/or nutrition, making the true denominator much lower.

There were 344 complete responses (estimated response rate 19%), and 115 (33%) were from PIBDNet. A total of 182 (53%) were from North America, most of whom (72%) were from a university-based practice, with 53% (182 of all 344 respondents) relatively new to practice (Table 1). Respondents had a median of 40 IBD patients in their practice, representing a median of 20% of their total number of patients seen. However, there were 84/344 (24%) for whom IBD patients made up the majority (>50%) of their practice. There was a predominance (median 60%) of patients with CD.

### 3.1. Crohn's Disease

Respondents reported a median of 40% (IQR 25-60%) of their CD patients to be treated with anti-TNF therapy. This was higher in North America (median 50% vs. 30% Europe vs. 30% elsewhere, p<0.001) (Table 2). IFX was most frequently the initial anti-TNF drug used for both luminal (median 90% (IQR 50-100%)) and perianal fistulizing CD (median 100% (IQR 90-100%)).

North Americans more often (80% vs. 38% Europe vs. 30% elsewhere, p<0.001) used anti-TNF therapy without a trial of IM (Table 2). However, they used IFX (but not ADA) in combination with concomitant IM less often in both IM-naïve patients (median 50% of time vs. 90% Europe vs. 100% elsewhere, p<0.001) and after IM failure (median 80% of time vs. 98% Europe vs. 90% elsewhere, p<0.001). For both IFX and ADA, North Americans were less likely to choose thiopurines as their combination IM (24% vs. 94% Europe vs. 79% elsewhere for IFX, p<0.001; 20% North America vs. 93% Europe vs. 69% elsewhere for IM-naïve on ADA, p<0.001; and 25% North America vs. 76% Europe vs. 68% elsewhere for ADA used after IFX secondary loss of response, p<0.001) but were likely to continue combination therapy for a longer period of time.

North Americans were less likely than physicians elsewhere in the world to strictly adhere to standard IFX induction protocols (42% vs. 66% Europe vs. 84% elsewhere, p<0.001), although similar ADA induction protocols were followed (Table 2). Respondents from all regions had similar approaches to regimen intensification, with most preferring to increase the dose of IFX and/or shorten the dosing interval depending on the individual patient (64%), rather than uniformly increasing the dose (27%) or shortening the interval (9%).

Almost half of respondents (45%) had ever stopped anti-TNF therapy for a patient with luminal CD in continuous remission, and this was more common in Europe (59% vs. 38% North America vs. 42% elsewhere, p=0.003). North Americans were also more likely to initiate anti-TNF therapy as early preventive therapy after an ileal resection for internal penetrating disease (median 80% (IQR 40-100%) vs. 50% (IQR 0-100%) in both Europe and elsewhere, p=0.011).

We also compared the practice of respondents from Canada and the USA (Supplemental Table 1). Canadians treated a smaller percentage of patients with CD with anti-TNF therapy (median 36% vs. 50%, p=0.004) and used more combination IM therapy with IFX (median 85% vs. 30%, p=0.001 for IM-naïve, median 98% vs. 75%, and p= 0.027 after IM failure) and ADA (median 50% vs. 20%; p=0.043 for IM- and IFX-naïve and median 100% vs. 70%; p=0.046 after IFX loss of response).

Comparing those in university-based practice to those in community-practice (Supplemental Table 2), there were few significant differences. Those in university-based practice more often used combination IM for IFX (median 80% vs. 25%, p=0.003 for IM-naïve patients, median 90% vs. 75%, and p=0.027 after IM failure). They also used combination IM more frequently with ADA, but only after loss of response to IFX (median 90% vs. 50%, p=0.009).

Finally, there were no significant differences between those new to practice (<10 years after fellowship training) compared to others (≥10 years after training) (Supplemental Table 3).

### 3.2. Ulcerative Colitis

UC patients were less often treated with anti-TNF therapy compared to patients with CD (median 10% (IQR 5-25%) vs. median 40% (IQR 25-60%), p<0.001). As with CD, anti-TNF use was higher in North America (median 20% of UC treated with anti-TNF therapy vs. 10% Europe vs. 5% elsewhere, p<0.001) (Table 3). Some respondents (15% overall) restricted their use of anti-TNF therapy to hospitalized, steroid-refractory UC patients. This was more frequent outside of North America (10% vs. 18% Europe vs. 28% elsewhere, p=0.021). Infliximab was most often used as the first anti-TNF agent (median 100% (IQR 80-100%)), and this did not vary by geography (p=0.735).

North Americans sometimes used anti-TNF therapy for patients with UC more frequently without a preceding trial of IM (76% vs. 47% Europe vs. 36% elsewhere, p<0.001) and more frequently as monotherapy (Table 3). However, similar to practice patterns with CD, North Americans less often used thiopurines (35% vs. 100% Europe vs. 86% elsewhere, p<0.001) and more often continued combination IM indefinitely (44% vs. 15% Europe vs. 29% elsewhere, p=0.004).

Standard IFX induction protocols were strictly followed less often by North Americans than colleagues elsewhere for both steroid-refractory (46% vs. 73% Europe vs. 61% elsewhere, p=0.002) and steroid-dependent patients (70% vs. 90% Europe vs. 78% elsewhere, p=0.002) (Table 3). North Americans were more likely to increase induction doses to 10 mg/kg for steroid-refractory disease (40% vs. 18% Europe vs. 17% elsewhere, p<0.001). Europeans were less likely to increase to 10 mg/kg for steroid-dependent patients (8% vs. 25% North America vs. 22% elsewhere, p=0.046).

One-third of respondents (33%) had stopped anti-TNF therapy for a UC patient in continuous remission, and this was more common in Europe (51% vs. 26% North America vs. 22% elsewhere, p<0.001). For UC patients with secondary loss of response to IFX due to antibody formation, respondents have gone on to use alternative anti-TNF therapies a median of 20% (IQR 0-91%) of the time. This was significantly more common in Europe (50% (IQR 0-100%) vs. 20 % (IQR 0-93%) North America vs. 0% (IQR 0-10%) elsewhere, p=0.001).

In comparing respondents from Canada to those in the USA (Supplemental Table 4), Canadians treated a larger percentage of patients with UC with combination therapy in both the IM-naïve (median 90% vs. 45%, p=0.004) and IM failure settings (median 100% vs. 70%, p=0.006). Canadians also less often adhered to standard IFX induction regimens (13% vs. 45%, p=0.001 for steroid-refractory disease, 31% vs. 55%, p=0.016 for steroid-dependent disease).

Similar to CD, the only difference between those in university-based practice compared to those in community-based practice was the frequency of combination IM use (Supplemental Table 5). Those in university-based practice used IM more frequently for IM-naïve patients (median 75% vs. 40%, p=0.027) but the difference for patients after IM failure did not reach statistical significance (median 90% vs. 75%, p=0.070).

Those with ≥10 years of experience after fellowship had more patients on anti-TNF therapy (median 15% (IQR 5–30) vs. 10 % (IQR 3–20), p = 0.010). (Supplemental Table 6).

### 3.3. Routine Testing

The vast majority of respondents (90%) performed some kind of routine testing before starting anti-TNF therapy. More testing was done in Europe (95%) compared to North America (90%) and elsewhere (83%) (p=0.039). Some type of testing for tuberculosis was performed by 90% overall (90% in North America vs. 95% Europe vs. 83% elsewhere, p=0.039. Chest X-ray was done relatively infrequently in North America (38% vs. 81% Europe vs. 76% elsewhere, p<0.001). QuantiFERON gold testing was more common in Europe (75% vs. 53% North America vs. 38% elsewhere, p<0.001). Rates of tuberculin skin testing were similar in all areas (68% North America vs. 57% Europe vs. 57% elsewhere, p=0.101). Once patients started on anti-TNF therapy, annual TB screening was more often done by North Americans (39% vs. 14 % Europe vs. 16% elsewhere, p<0.001). A majority of respondents in all locations routinely check varicella (54% North America vs. 59% Europe vs. 50% elsewhere, p=0.500) and Hepatitis B serology (72% North America vs. 70% Europe vs. 64% elsewhere, p=0.482). Epstein-Barr virus titres were checked less often in North America (25% vs. 61% Europe vs. 38% elsewhere, p<0.001).

Performance of some type of tuberculosis screening was similar across North America (94% Canada vs. 90% USA, p=0.447). However, compared to respondents from the USA, Canadians were more likely to perform TB skin testing (91% vs. 64%, p=0.003) and chest X-ray (84% vs. 29%, p<0.001). However, Canadians less often performed QuantiFERON gold testing (9% vs. 63%, p<0.001). Canadians more often checked varicella serology (78% vs. 49%, p=0.003) and EBV titres (50% vs. 19%, p<0.001). Respondents from the USA performed routine TB testing in follow-up frequently compared to Canadians (47% vs. 6%, p<0.001).

University-based respondents generally performed screening tests more frequently. TB screening was requested routinely by 93% of university-based respondents, compared to 83% of their community counterparts (p=0.009). Tuberculin skin testing and QuantiFERON gold testing rates were similar between groups, but chest X-rays were more common in the university setting (63% vs. 42%, p<0.001). Varicella screening and EBV screening were both more common in the university-based practices (59% vs. 46%, p=0.035; 42% vs. 26%, p=0.005, respectively).

Those newer to practice (<10 years after fellowship) were more likely to perform QuantiFERON gold testing (63% vs. 52%, p=0.040). All other preinduction testing and ongoing monitoring were similar between groups.

### 3.4. Access Limitations

The percentages of respondents reporting being limited in their use of anti-TNF therapy were 36% for CD and 37% for UC. However, perceived restriction of use was much more common in areas outside of North America or Europe. This was true for almost all scenarios for patients with CD and UC presented to respondents (Table 4).

When examining those within North America, Canadians generally identified more access restrictions (Supplemental Table 7). Canadians reported more often being limited in their use of anti-TNF therapy in newly diagnosed patients with CD (41% vs. 19% USA, p=0.009). They also reported more difficulty initiating anti-TNF therapy in patients with steroid-dependent UC without first trying an immunomodulator (41% vs. 29%, p=0.007). Lastly, Canadians more often reported not being able to use their desired dosage (31% vs. 15%, p=0.025).

The only difference between those in university-based vs. community-based practices (Supplemental Table 8) was decreased limitation for UC in university-based practices (59% vs. 73% never limited, p=0.029).

Those new to practice (<10 years following fellowship) reported more frequently being limited in their access to anti-TNF therapy for newly diagnosed CD patients (24% vs. 15%, p=0.026).

## 4. Discussion

Pediatric IBD specialists worldwide are attempting within PIBDNet to facilitate collaborative studies which will guide optimal pediatric care of children with CD and UC. Our PIBDNet survey of practice patterns with respect to anti-TNF use identifies significant differences between pediatric gastroenterologists in North America, Europe, and elsewhere. Identifying and characterizing these differences is the first step in studying their effect on outcomes of interest.

Compared to colleagues elsewhere, North American pediatric gastroenterologists treat a higher percentage of pediatric CD and UC with anti-TNF antibodies and more often prescribe anti-TNF agents without a prior trial of IM. The optimal positioning of anti-TNF therapy relative to other therapies in both CD and UC is still debated. Our North American pediatric data are very different from adult surveys of the American Gastroenterological Association in 2009 and of gastroenterologists in Maryland and Washington, DC in 2007, where 70% and 48% of respondents, respectively, said they would use IM prior to prescribing IFX. [9, 10]. Similarly, 90% of Swiss adult gastroenterologists reported that they use a conventional step-up strategy, first prescribing thiopurines before using anti-TNF therapy [11].

In all parts of the world pediatric CD was reported to be more often treated with anti-TNF therapy compared to UC. Similarly, the Canadian Child Inflammatory Bowel Disease Network has reported that 61% of patients with CD are maintained on an anti-TNF therapy, compared with 31% of UC patients [12].

In this survey we found IFX to be the overwhelming first choice anti-TNF drug for both CD and UC, which is consistent with previous adult reports and our clinical experience [11, 13]. What drives this choice is unclear. Others have cited the ability to monitor patients regularly and increased familiarity with IFX given that it has been available for IBD treatment for longer [13].

North American respondents (especially in the USA) reported using anti-TNF antibodies as monotherapy more often than their global counterparts, perhaps in part because of the dominant practice elsewhere of adding anti-TNF in patients already receiving but responding unsatisfactorily to IM. The common initiation in North America of infliximab as monotherapy is concerning, given the now substantial body of evidence, including adult randomized controlled trial and pediatric observational data, documenting the importance of concomitant IM in reducing the likelihood of secondary loss of responsiveness related to anti-infliximab antibodies [5, 14, 15].

Choice of combination IM was also different between North America and elsewhere, for both CD and UC. There is no prospective study directly comparing thiopurine to methotrexate combination therapy for any anti-TNF drug for either CD or UC. The SONIC study demonstrated improvement in multiple outcomes comparing adults treated with IFX and azathioprine combination therapy to those treated with either therapy alone [14]. The adult COMMIT study, whilst not demonstrating a treatment benefit to combination therapy with methotrexate, did demonstrate less immunogenicity and higher drug levels at trough [16]. In our sample, North Americans preferred methotrexate, whereas practitioners elsewhere more often chose thiopurines. The reasons underlying this difference are unknown. Certainly, the risk of hepatocellular T-cell lymphoma associated with use of thiopurines is well known. The same risk has not been associated with methotrexate in patients with IBD [17]. As recently reported, data from the prospective DEVELOP safety registry of pediatric IBD demonstrated an increased incidence of malignancy compared to age- and gender-matched healthy controls in the SEER database in children with IBD treated with thiopurines with or without biologics [18]. However, the use of methotrexate is presently not evidence-based and thus carries a slightly lower recommendation than thiopurines by the European Society for Pediatric Gastroenterology, Hepatology and Nutrition (ESPGHAN) [19].

Our respondents, especially from North America, preferred to continue combination IM for longer when used with IFX, for both CD and UC patients. Although there are adult data to support significant benefit of combination azathioprine with IFX for CD up to 12 months [14], no such prospective evidence exists to specifically guide pediatric practice. ESPGHAN and European Crohn's and Colitis Organization (ECCO) joint guidelines suggest discontinuing combination IM therapy after 6 months for CD and 4-8 months for UC, especially in males and those who are not at high risk for a severe disease course [19, 20].

Standard induction therapy with IFX was less often adhered to by North Americans. This difference was largest in CD but was also observed in steroid-refractory and steroid-dependent UC. Recognition of the need for customized dosing of IFX and ADA is not new. Escalating IFX doses beyond 5 mg/kg every 8 weeks in the setting of unsatisfactory response has been practised for years, especially in the adult population [21] and has also been reported in pediatric practice [5]. Similar practice with ADA (prescribing more than 40 mg every 2 weeks) has also been described in both adult and pediatric IBD populations [22, 23]. Current guidelines for management of children with IBD also endorse this practice [19, 20].

More recently, an intensified IFX induction regimen, with 3 doses given over a median of 24 days (instead of the usual 6 weeks), was studied for adult patients with acute severe UC [24]. These investigators found significantly decreased risk of colectomy during induction therapy. Many adult gastroenterologists are already practicing this way. A survey of 123 members from Crohn's and Colitis Foundation of America Clinical Research Alliance and the International Organization for Inflammatory Bowel Disease demonstrated that only 24% of respondents used standard IFX induction dosing for severe UC [25]. Individualized dosing based on therapeutic drug monitoring (TDM) seems logical, but prospective controlled trials confirming efficacy of TDM are hitherto lacking and access to TDM remains inconsistent in clinical practice [10].

Most respondents reported performing some screening investigations prior to starting anti-TNF therapy. However, considering the well-known risk of tuberculosis with anti-TNF therapy [26], it is surprising that only 90% screened for tuberculosis. This rate was lower in North America compared with Europe. The reasons for this are unclear, although it may be related to the very low prevalence of tuberculosis infection in this part of the world [27]. Pediatric guidelines for Crohn's disease from ECCO and ESPGHAN are clear that testing for tuberculosis prior to starting an anti-TNF agent should by mandatory [19]. They also suggest screening for hepatitis B virus and varicella zoster virus immunity, with consideration of immunization in those at risk of infection prior to starting anti-TNF therapy. The high rate of yearly TB testing during follow-up was surprising, since this does not appear to be based on guideline recommendations.

Our study's results should be interpreted with some limitations in mind. Respondents most likely answered our questions based on recall, rather than a detailed audit of their actual practice. We had 344 respondents who completed the entire survey, for a conservative estimated response rate of 19%. However, many of the 1800 potential respondents from the Pediatric GI Bulletin Board likely do not treat pediatric IBD (they may be hepatologists, nutritionists, surgeons, nurses, dieticians, etc.), making a realistic response rate much higher. A systematic review and meta-analysis of general practitioner response rates to postal surveys estimated an average response rate of 61% (95% CI 59%-63%) [28]. Email surveys have been shown to be less effective than postal surveys, with 1.82 times lower odds of response [29]. Taken together, these data suggest that our response rate of 19% is acceptable. Another limitation is the low response rate from Asia, Oceania, Africa, and South America, limiting our study's generalizability. This is particularly important when interpreting access limitations, as these parts of the world likely have the most inadequate access to anti-TNF therapy. Also, our investigation of the reasons behind the reported differences in practice, including access limitations, was brief in order to keep the survey length manageable for respondents. Elucidating the reasons behind the differences reported here is an important area for future study.

The use of anti-TNF therapies is significantly different in North America compared to Europe and other parts of the world. In North America, anti-TNF therapy is used earlier and more aggressively. Combination therapy with an IM is less common, and when it is used, thiopurines are avoided.

## Figures and Tables

**Table 1:** Demographics for the 344 respondents who completed the survey. Results are presented as N (%) or median (interquartile range) as appropriate.

Characteristic	N=344
Continent	
North America	183 (53%)
Europe	103 (30%)
Asia	20 (6%)
Oceania	21 (6%)
Africa	10 (3%)
South America	7 (2%)
Primary practice type	
University-based	248 (72%)
Community-based	96 (28%)
Years in practice following fellowship	
<10	184 (53%)
10-20	92 (27%)
20-40	68 (20%)
Percentage of practice devoted to IBD	20 (10-40)
Number of IBD patients in practice	40 (20-90)
Percentage of patients with CD	60 (50-70)
Oldest IBD patient ever treated (years)	
≤18	147 (43%)
18-21	93 (27%)
21-25	82 (24%)
25-75	21 (6%)

IBD: inflammatory bowel disease; CD: Crohn's disease.

**Table 2:** Practice patterns in luminal Crohn's disease with infliximab (IFX) and adalimumab (ADA). Results are presented as percentages or median (interquartile range) as appropriate.

		North America	Europe	Elsewhere	p-value
		N=183	N=103	N=58	
Percentage of patients treated with anti-TNF therapy		50 (30-65)	30 (20-50)	30 (20-50)	<0.001
Anti-TNF sometimes used without IM trial		80%	38%	30%	<0.001
Percentage of time combination IM used with anti-TNF					
IFX	IM-naïve	50 (10-90)	90 (20-100)	100 (60-100)	<0.001
	After IM failure	80 (40-100)	98 (68-100)	90 (50-100)	0.003
ADA	IM- and IFX-naïve	25 (0-90)	80 (10-100)	20 (0-90)	0.056
	after IFX LoR	75 (10-100)	90 (20-100)	55 (0-100)	0.510
Thiopurine always or usually chosen as combination IM					
IFX		24%	94%	79%	<0.001
ADA	IM-naïve	20%	93%	69%	<0.001
	after IFX LoR	25%	76%	68%	<0.001
Combination IM continued indefinitely					
IFX		48%	16%	32%	<0.001
ADA	IM-naïve	49%	14%	11%	<0.001
	after IFX LoR	54%	14%	27%	<0.001
Always adhere to standard induction					
IFX		42%	66%	84%	<0.001
ADA		66%	64%	70%	0.932

anti-TNF: anti-tumour necrosis factor; IM: immunomodulator; LoR: loss of response.

**Table 3:** Practice patterns in ulcerative colitis with infliximab. Results are presented as percentages or median (interquartile range) as appropriate.

	North America	Europe	Elsewhere	p-value
	N=183	N=103	N=58	
Percentage of the time Anti-TNF used	20 (5-30)	10 (5-23)	5 (2-10)	<0.001
Anti-TNF sometimes used without IM trial				
Steroid-dependent	76%	47%	36%	<0.001
Percentage of time combination IM used with anti-TNF				
IM-naïve	50 (0-90)	85 (15-100)	90 (25-100)	0.002
After IM-failure	80 (20-100)	95 (50-100)	100 (50-100)	0.003
Thiopurine always or usually chosen as combination IM	35%	100%	86%	<0.001
Combination IM continued indefinitely	44%	15%	29%	0.004
Always adhere to standard induction				
Steroid-refractory	46%	73%	61%	0.002
Steroid-dependent	70%	90%	78%	0.002

**Table 4:** Access limitations identified by respondents. Results are presented as N (%).

	North America	Europe	Elsewhere	p-value
	N=183	N=103	N=58	
Access never limited for CD	103 (56%)	79 (77%)	16 (28%)	<0.001
Access never limited for UC	99 (54%)	76 (74%)	18 (31%)	<0.001
Access is sometimes limited in newly diagnosed CD	35 (19%)	12 (12%)	19 (33%)	0.005
Access is sometimes limited before failure of steroids or EEN followed by IM in CD	42 (23%)	13 (13%)	29 (50%)	<0.001
Access is sometimes limited except in hospitalized, steroid-refractory UC	20 (11%)	9 (9%)	12 (21%)	0.067
Access is sometimes limited in steroid-dependent UC before failure of steroids followed by an immunomodulator	41 (22%)	12 (12%)	23 (40%)	<0.001
Dosing regimen is sometimes limited	32 (17%)	9 (9%)	20 (34%)	<0.001
Duration of anti-TNF therapy is sometimes limited	5 (3%)	8 (8%)	5 (9%)	0.083
Access to day clinics for infusion is sometimes limited	14 (8%)	5 (5%)	6 (10%)	0.418

## Data Availability

Requests for original data will be considered by the corresponding author.

## Supplementary Materials

Supplementary Materials Supplemental Table 1. Practice patterns in luminal Crohn's disease with infliximab (IFX) and adalimumab (ADA) comparing Canada and USA. Supplemental Table 2. Practice patterns in luminal Crohn's disease with infliximab (IFX) and adalimumab (ADA) comparing university-based and community-based practices. Supplemental Table 3. Practice patterns in luminal Crohn's disease with infliximab (IFX) and adalimumab (ADA) comparing those new to practice (<10 years after fellowship training) to others (≥10 years after training). Supplemental Table 4. Practice patterns in ulcerative colitis with infliximab comparing Canada and USA. Supplemental Table 5. Practice patterns in ulcerative colitis with infliximab comparing university-based and community-based practices. Supplemental Table 6. Practice patterns in ulcerative colitis with infliximab comparing those new to practice (<10 years after fellowship training) to others (≥10 years after training). Supplemental Table 7. Access limitations identified by respondents comparing Canada and USA. Supplemental Table 8. Access limitations identified by respondents comparing university-based and community-based practices. Supplemental Table 9. Access limitations identified by respondents comparing those new to practice (<10 years after fellowship training) to others (≥10 years after training).
