# Supplementary material for: The Continental Divide: Anti-TNF Use in Pediatric IBD Is Different in North America Compared to Other Parts of the World
Source: Can J Gastroenterol Hepatol. 2018 Jun 12;2018:3190548. doi: 10.1155/2018/3190548 (PMC6020509; doi:10.1155/2018/3190548)
Supplement: Supplementary Materials — Supplemental Table 1. Practice patterns in luminal Crohn's disease with infliximab (IFX) and adalimumab (ADA) comparing Canada and USA. Supplemental Table 2. Practice patterns in luminal Crohn's disease with infliximab (IFX) and adalimumab (ADA) comparing university-based and community-based practices. Supplemental Table 3. Practice patterns in luminal Crohn's disease with infliximab (IFX) and adalimumab (ADA) comparing those new to practice (<10 years after fellowship training) to others (≥10 years after training). Supplemental Table 4. Practice patterns in ulcerative colitis with infliximab comparing Canada and USA. Supplemental Table 5. Practice patterns in ulcerative colitis with infliximab comparing university-based and community-based practices. Supplemental Table 6. Practice patterns in ulcerative colitis with infliximab comparing those new to practice (<10 years after fellowship training) to others (≥10 years after training). Supplemental Table 7. Access limitations identified by respondents comparing Canada and USA. Supplemental Table 8. Access limitations identified by respondents comparing university-based and community-based practices. Supplemental Table 9. Access limitations identified by respondents comparing those new to practice (<10 years after fellowship training) to others (≥10 years after training). [file 3190548.f1.pdf]

SUPPLEMENTAL TABLES

Supplemental Table 1. Practice patterns in luminal Crohn's disease with infliximab (IFX) and adalimumab (ADA) comparing Canada and USA. Results are presented as percentages or median (interquartile range) as appropriate.

			Canada N=32	USA N=150	p-value
Percentage of patients treated with anti-TNF therapy			36 (25-50)	50 (30-65)	0.004
Anti-TNF sometimes used without IM trial			84%	79%	0.464
Percentage of time combination IM used with anti-TNF					
	IFX	IM-naïve	85 (50-100)	30 (1-80)	0.001
		After IM failure	98 (50-100)	75 (30-100)	0.027
	ADA	IM- and IFX-naïve	50 (20-100)	20 (0-85)	0.043
		after IFX LoR	100 (35-100)	70 (5-100)	0.046
Thiopurine always or usually chosen as combination IM					
	IFX		15%	26%	0.209
	ADA	IM-naïve	13%	23%	0.331
		after IFX LoR	18%	26%	0.491
Combination IM continued indefinitely					
	IFX		52%	47%	0.611
	ADA	IM-naïve	58%	46%	0.494
		after IFX LoR	64%	52%	0.697
Always adhere to standard induction					
	IFX		38%	43%	0.494
	ADA		50%	70%	0.091

Supplemental Table 2. Practice patterns in luminal Crohn's disease with infliximab (IFX) and adalimumab (ADA) comparing university-based and community-based practices. Results are presented as percentages or median (interquartile range) as appropriate.

			Community N=96	University N=248	p-value
Percentage of patients treated with anti-TNF therapy			40 (25-60)	40 (20-60)	0.116
Anti-TNF sometimes used without IM trial			53%	61%	0.215
Percentage of time combination IM used with anti-TNF					
	IFX	IM-naïve	25 (0-100)	80 (20-100)	0.003
		After IM failure	75 (20-100)	90 (50-100)	0.027
	ADA	IM- and IFX-naïve	20 (0-80)	50 (0-95)	0.123
		after IFX LoR	50 (0-90)	90 (20-100)	0.009
Thiopurine always or usually chosen as combination IM					
	IFX		54%	55%	0.534
	ADA	IM-naïve	50%	45%	0.630
		after IFX LoR	47%	42%	0.711
Combination IM continued indefinitely					
	IFX		32%	37%	0.828
	ADA	IM-naïve	29%	36%	0.488
		after IFX LoR	40%	42%	0.920
Always adhere to standard induction					
	IFX		54%	57%	0.433
	ADA		67%	65%	0.792

Supplemental Table 3. Practice patterns in luminal Crohn's disease with infliximab (IFX) and adalimumab (ADA) comparing those new to practice (<10 years after fellowship training) to others (≥10 years after training). Results are presented as percentages or median (interquartile range) as appropriate.

			<10 years N=166	≥10 years N=178	p-value
Percentage of patients treated with anti-TNF therapy			43 (25-60)	40 (25-50)	0.154
Anti-TNF sometimes used without IM trial			58%	59%	0.915
Percentage of time combination IM used with anti-TNF					
	IFX	IM-naïve	70 (18-100)	75 (10-100)	0.800
		After IM failure	90 (50-100)	90 (50-100)	0.592
	ADA	IM- and IFX-naïve	50 (0-90)	50 (0-93)	0.599
		after IFX LoR	90 (28-100)	60 (0-100)	0.205
Thiopurine always or usually chosen as combination IM					
	IFX		52%	58%	0.282
	ADA	IM-naïve	43%	49%	0.312
		after IFX LoR	38%	48%	0.105
Combination IM continued indefinitely					
	IFX		32%	39%	0.476
	ADA	IM-naïve	28%	42%	0.265
		after IFX LoR	37%	45%	0.875
Always adhere to standard induction					
	IFX		52%	60%	0.414
	ADA		60%	71%	0.113

Supplemental Table 4. Practice patterns in ulcerative colitis with infliximab comparing Canada and USA.

Results are presented as percentages or median (interquartile range) as appropriate.

	Canada N=32	USA N=150	p-value
Percentage of the time Anti-TNF used	20 (8-50)	15 (5-30)	0.446
Anti-TNF sometimes used without IM trial			
Steroid-dependent	66%	78%	0.567
Percentage of time combination IM used with anti-TNF			
IM-naïve	90 (20-100)	45 (0-80)	0.004
After IM-failure	100 (75-100)	70 (20-100)	0.006
Thiopurine always or usually chosen as combination IM	21%	39%	0.209
Combination IM continued indefinitely	52%	42%	0.506
Always adhere to standard induction			
Steroid-refractory	13%	45%	0.001
Steroid-dependent	31%	55%	0.016

Supplemental Table 5. Practice patterns in ulcerative colitis with infliximab comparing university-based and community-based practices. Results are presented as percentages or median (interquartile range) as appropriate.

	Community N=96	University N=248	p-value
Percentage of the time Anti-TNF used	10 (3-30)	11 (5-25)	0.384
Anti-TNF sometimes used without IM trial			
Steroid-dependent	69%	57%	0.198
Percentage of time combination IM used with anti-TNF			
IM-naïve	40 (0-98)	75 (10-100)	0.027
After IM-failure	75 (23-100)	90 (50-100)	0.070
Thiopurine always or usually chosen as combination IM	60%	66%	0.213
Combination IM continued indefinitely	32%	32%	0.782
Always adhere to standard induction			
Steroid-refractory	55%	47%	0.160
Steroid-dependent	60%	62%	0.827

Supplemental Table 6. Practice patterns in ulcerative colitis with infliximab comparing those new to practice (<10 years after fellowship training) to others (≥10 years after training). Results are presented as percentages or median (interquartile range) as appropriate.

	<10 years N=166	≥10 years N=178	p-value
Percentage of the time Anti-TNF used	15 (4-25)	10 (5-25)	0.395
Anti-TNF sometimes used without IM trial			
Steroid-dependent	35%	56%	0.308
Percentage of time combination IM used with anti-TNF			
IM-naïve	50 (8-100)	75 (0-100)	0.443
After IM-failure	80 (50-100)	90 (50-100)	0.682
Thiopurine always or usually chosen as combination IM	58%	70%	0.025
Combination IM continued indefinitely	28%	36%	0.069
Always adhere to standard induction			
Steroid-refractory	49%	49%	0.905
Steroid-dependent	60%	63%	0.532

Supplemental Table 7. Access limitations identified by respondents comparing Canada and USA.

	Canada N=32	USA N=150	p-value
Access never limited for CD	50%	65%	0.128
Access never limited for UC	41%	64%	0.023
Access is sometimes limited in newly diagnosed CD	31%	17%	0.057
Access is sometimes limited before failure of steroids or EEN followed by IM in CD	41%	19%	0.009
Access is sometimes limited except in hospitalized, steroid-refractory UC	16%	10%	0.356
Access is sometimes limited in steroid-dependent UC before failure of steroids followed by an immunomodulator	41%	19%	0.007
Dosing regimen is sometimes limited	31%	15%	0.025
Duration of anti-TNF therapy is sometimes limited	6%	2%	0.182
Access to day clinics for infusion is sometimes limited	9%	7%	0.694

Supplemental Table 8. Access limitations identified by respondents comparing university-based and community-based practices.

	Community N=96	University N=248	p-value
Access never limited for CD	70%	61%	0.164
Access never limited for UC	73%	59%	0.029
Access is sometimes limited in newly diagnosed CD	17%	20%	0.460
Access is sometimes limited before failure of steroids or EEN followed by IM in CD	18%	27%	0.071
Access is sometimes limited except in hospitalized, steroid-refractory UC	12%	12%	0.870
Access is sometimes limited in steroid-dependent UC before failure of steroids followed by an immunomodulator	17%	24%	0.131
Dosing regimen is sometimes limited	14%	19%	0.205
Duration of anti-TNF therapy is sometimes limited	4%	5%	0.581
Access to day clinics for infusion is sometimes limited	5%	8%	0.360

Supplemental Table 9. Access limitations identified by respondents comparing those new to practice (<10 years after fellowship training) to others (≥10 years after training).

	<10 years N=166	≥10 years N=178	p-value
Access never limited for CD	60%	67%	0.187
Access never limited for UC	59%	65%	0.264
Access is sometimes limited in newly diagnosed CD	24%	15%	0.026
Access is sometimes limited before failure of steroids or EEN followed by IM in CD	27%	22%	0.262
Access is sometimes limited except in hospitalized, steroid-refractory UC	12%	12%	0.943
Access is sometimes limited in steroid-dependent UC before failure of steroids followed by an immunomodulator	41%	35%	0.261
Dosing regimen is sometimes limited	18%	17%	0.873
Duration of anti-TNF therapy is sometimes limited	5%	6%	0.740
Access to day clinics for infusion is sometimes limited	8%	6%	0.421
